# Comparison of *In Vitro* Activity of Ceftazidime-Avibactam and Imipenem-Relebactam against Clinical Isolates of Pseudomonas aeruginosa

**DOI:** 10.1128/spectrum.00932-23

**Published:** 2023-05-18

**Authors:** Leilei Wang, Xuefei Zhang, Xun Zhou, Fan Yang, Qinglan Guo, Minggui Wang

**Affiliations:** a Institute of Antibiotics, Huashan Hospital, Fudan University, Shanghai, China; b Key Laboratory of Clinical Pharmacology of Antibiotics, National Heath Commission of People’s Republic of China, Shanghai, China; Taichung Veterans General Hospital

**Keywords:** *Pseudomonas aeruginosa*, ceftazidime-avibactam, imipenem-relebactam, *in vitro* activity

## Abstract

The role of novel β-lactam/β-lactamase inhibitor combinations in ceftazidime-nonsusceptible (CAZ-NS) and imipenem-nonsusceptible (IPM-NS) Pseudomonas aeruginosa has not been fully elucidated. This study evaluated the *in vitro* activity of novel β-lactam/β-lactamase inhibitor combinations against Pseudomonas aeruginosa clinical isolates, determined how avibactam restored ceftazidime activity, and compared the activity of ceftazidime-avibactam (CZA) and imipenem-relebactam (IMR) against KPC-producing P. aeruginosa. Similar high susceptibility rates for CZA, IMR, and ceftolozane-tazobactam (88.9% to 89.8%) were found for 596 P. aeruginosa clinical isolates from 11 hospitals in China, and a higher susceptibility rate to ceftazidime than imipenem was observed (73.5% versus 63.1%). For CAZ-NS and IPM-NS isolates, susceptibility rates for CZA, ceftolozane-tazobactam, and IMR were 61.5% (75/122), 54.9% (67/122), and 51.6% (63/122), respectively. For CAZ-NS, IPM-NS but CZA-susceptible isolates, 34.7% (26/75) harbored acquired β-lactamases with KPC-2 predominant (*n* = 19), and 45.3% (34/75) presented overexpression of chromosomal β-lactamase *ampC*. Among 22 isolates carrying KPC-2 carbapenemase alone, susceptibility rates to CZA and IMR were 86.4% (19/22) and 9.1% (2/22), respectively. Notably, 95% (19/20) of IMR-nonsusceptible isolates had an inactivating mutation of *oprD* gene. In conclusion, CZA, ceftolozane-tazobactam, and IMR exhibit high activity against P. aeruginosa, and CZA is more active than IMR against CAZ-NS and IPM-NS isolates as well as KPC-producing P. aeruginosa. Avibactam overcomes ceftazidime resistance engendered by KPC-2 enzyme and overexpressed AmpC.

**IMPORTANCE** The emergence of antimicrobial resistance poses a particular challenge globally, and the concept of P. aeruginosa with “difficult-to-treat” resistance (DTR-P. aeruginosa) was proposed. Here, P. aeruginosa clinical isolates were highly susceptible to three β-lactamase inhibitor combinations, CZA, IMR, and ceftolozane-tazobactam. The combination of KPC-2 enzyme and nonfunctional porin OprD contributed to IMR resistance in P. aeruginosa, and CZA was more active than IMR in fighting against KPC-2-producing P. aeruginosa. CZA also showed good activity against CAZ-NS and IPM-NS P. aeruginosa, primarily by inhibiting KPC-2 enzyme and overproduced AmpC, supporting the clinical use of CZA in the treatment of infections caused by DTR-P. aeruginosa.

## INTRODUCTION

Pseudomonas aeruginosa is a major cause of serious infections in humans, which often become notoriously refractory to treatment and lead to considerable morbidity, mortality, and socioeconomic impacts worldwide. In this scenario, β-lactam antibiotics remain one of the mainstays of therapy. One interesting phenomenon is the higher *in vitro* susceptibility rate of this pathogen to ceftazidime than to carbapenems (1). Ceftazidime, carbapenems, and piperacillin-tazobactam demonstrated comparable antipseudomonal roles as monotherapies for P. aeruginosa bacteremia in terms of mortality, clinical and microbiological outcomes, and adverse events (2).

The emergence of antimicrobial resistance poses a particular challenge globally, and the concept of P. aeruginosa with “difficult-to-treat” resistance (DTR-P. aeruginosa) was proposed in 2018 (3). DTR-P. aeruginosa exhibited nonsusceptibility to β-lactams (piperacillin-tazobactam, ceftazidime, cefepime, imipenem-cilastatin, meropenem, and aztreonam) and fluoroquinolones (ciprofloxacin and levofloxacin) (4). Mechanisms of resistance to β-lactams for P. aeruginosa are complex and multifactorial, including β-lactamases, porin, and efflux pump. Additionally, resistance mechanisms vary for imipenem and ceftazidime. Acquired β-lactamases are relatively uncommon in P. aeruginosa, except in local outbreaks, but are usually diverse (5). Mutational inactivation of the carbapenem-specific porin OprD is a leading contributor to resistance to imipenem (6). Chromosomally encoded class C cephalosporinase AmpC (Pseudomonas-derived cephalosporinase, PDC β-lactamase) is commonly responsible for resistance to ceftazidime via structural mutations or overexpression. The intrinsic resistance-nodulation-division family of efflux systems (MexAB-OprM, MexCD-OprJ, MexEF-OprN, and MexXY-OprM) are also critical features associated with β-lactam resistance (7), and the MexAB-OprM tripartite efflux system was shown to use ceftazidime as a substrate (8).

Novel β-lactam/β-lactamase inhibitor combinations, such as ceftazidime-avibactam (CZA), ceftolozane-tazobactam (CT), and imipenem-cilastatin-relebactam (IMR), have recently been approved and cast a new spotlight on the treatment of multidrug-resistant (MDR) Gram-negative bacterial infections. CZA expands the therapeutic coverage of ceftazidime against P. aeruginosa strains with inhibitory Ambler class A, C, and some D β-lactamases. IMR exhibits an antimicrobial spectrum similar to that of CZA except for inhibition activity against OXA-48 enzyme. Ceftolozane, a novel oxyimino-aminothiazolyl cephalosporin, is characterized by its stability in PDC hydrolysis. In previous studies, CZA and CT showed excellent *in vitro* activity against P. aeruginosa with susceptibility rates of 86.5% and 88.5%, respectively, and IMR demonstrated a susceptibility rate of 90.8% (9, 10). These combinations are recommended for the treatment of infections caused by DTR-P. aeruginosa according to the guidance of the Infectious Diseases Society of America (IDSA) (4).

Recently, sporadic KPC-producing P. aeruginosa isolates are emerging in China. The resistant gene *bla*_KPC-2_ is commonly located in plasmids with mobile elements and is frequently detected in sequence type 463 (ST463) isolates (11). These *bla*_KPC-2_-carrying P. aeruginosa isolates displayed various levels of susceptibility to CZA, from 49.7% (75/151) to 100% (16/16) (11, 12). However, little information is available about their susceptibility to novel agents, including IMR.

This study investigated the *in vitro* activities of CZA, CT, and IMR against clinical P. aeruginosa isolates collected from multiple medical centers in China and uncovered the extent to which avibactam restored the activity of ceftazidime in ceftazidime-nonsusceptible (CAZ-NS) and imipenem-nonsusceptible (IPM-NS) P. aeruginosa. This study found high *in vitro* activities of CZA, CT, and IMR against P. aeruginosa clinical isolates. The combination of KPC-2 carbapenemase and nonfunctional porin OprD led to IMR resistance in P. aeruginosa, and CZA was more active than IMR in fighting against KPC-2-producing P. aeruginosa. CZA also retained good activity against CAZ-NS and IPM-NS P. aeruginosa isolates, as avibactam overcame the ceftazidime resistance engendered by acquired β-lactamase KPC-2 and overproduced chromosomal β-lactamase AmpC.

## RESULTS

### Susceptibility profile of the 596 P. aeruginosa isolates.

Overall, susceptibility rates to CZA, IMR, and CT were 89.8%, 88.9%, and 88.9%, respectively, among the 596 P. aeruginosa isolates. The MIC_50_ and MIC_90_ for CZA were 2 mg/L and 16 mg/L, respectively, and avibactam restored the susceptibility of ceftazidime in 61.4% (97/158) of CAZ-NS isolates. Moreover, higher susceptibility to CAZ than to IPM was observed (73.5% versus 63.1%; McNemar’s test, *P < *0.001). The most active agents against 596 P. aeruginosa isolates were colistin (susceptibility rate, 98.3%) and amikacin (90.8%), and susceptibility rates to the remaining six agents ranged from 58.2% (aztreonam) to 78.2% (cefepime) (Table 1). DTR-P. aeruginosa accounted for 11.9% (71/596).

**TABLE 1:** Activity of ceftazidime-avibactam, ceftolozane-tazobactam, imipenem-relebactam, and comparator antimicrobial agents against 596 P. aeruginosa clinical isolates

Antimicrobial agent^*a*^	MIC (mg/L)			Susceptibility (%)^*b*^		
	MIC_50_	MIC_90_	Range	S	I	R
Piperacillin	8	>128	1 to >128	70.6	12.8	16.6
Piperacillin-tazobactam	8	128	0.25 to >128	73.7	12.2	14.1
Ceftazidime	4	64	0.5 to >128	73.5	6.2	20.3
Ceftazidime-avibactam	2	16	0.25 to >128	89.8		10.2
Imipenem	2	32	0.25 to >128	63.1	2.0	34.9
Imipenem-relebactam	0.5	4	≤0.06 to >128	88.9	3.4	7.7
Ceftolozane-tazobactam	0.5	8	0.25 to >128	88.9	1.2	9.9
Cefepime	4	32	0.25 to >128	78.2	8.2	13.6
Aztreonam	8	128	0.25 to >128	58.2	13.8	28.0
Meropenem	1	32	≤0.06 to >128	62.8	6.0	31.2
Amikacin	4	16	1 to >128	90.8	3.7	5.5
Ciprofloxacin	0.25	16	≤0.06 to >128	71.8	5.2	23.0
Colistin	1	2	≤0.25 to >32	98.3		1.7

a Tazobactam, avibactam, and relebactam were tested at a fixed concentration of 4 mg/L.

b S, susceptible; I, intermediate; R, resistant.

**FIG 1:**
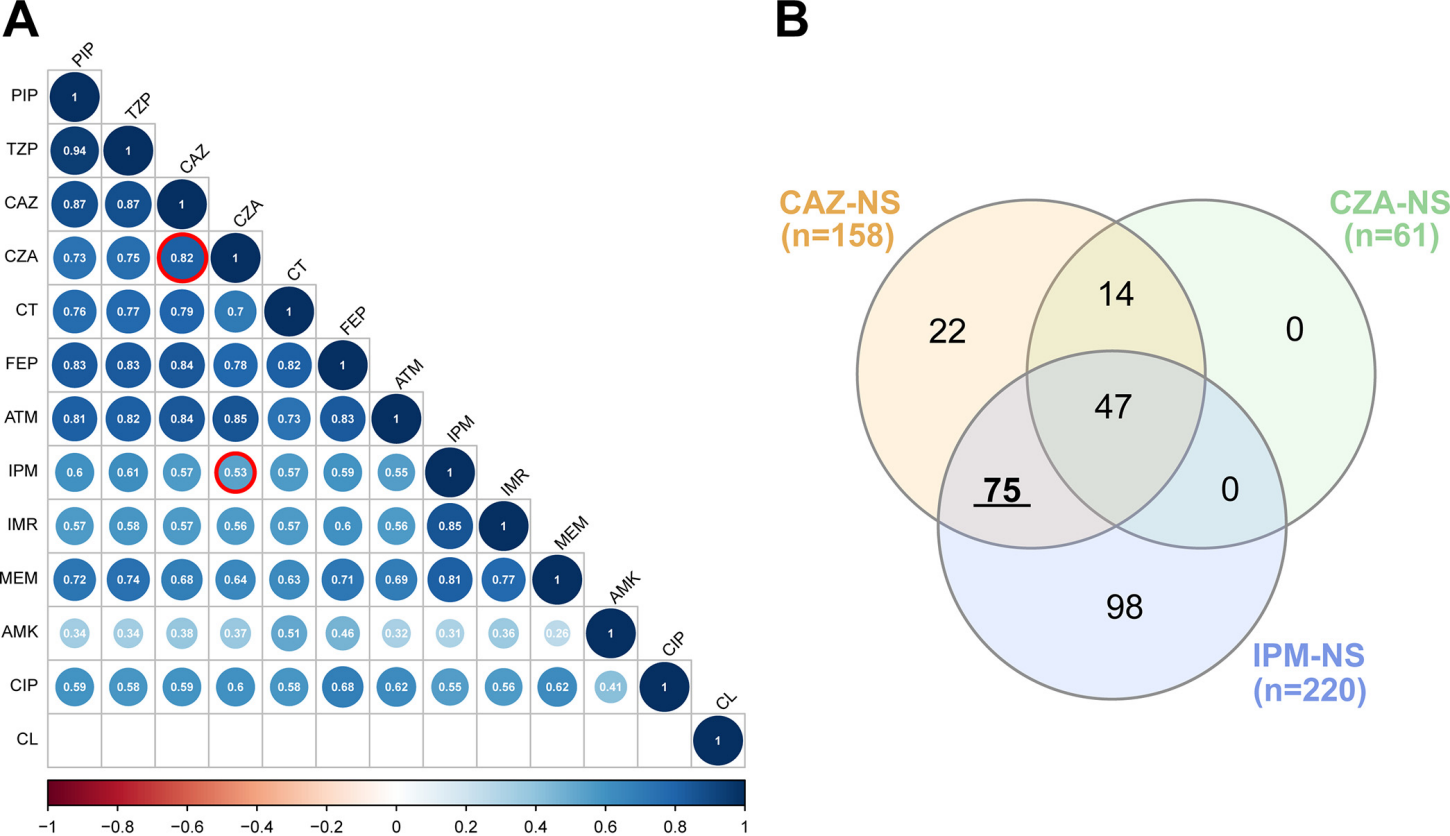
MIC correlation between different antimicrobials and nonsusceptibility overlap for Pseudomonas aeruginosa clinical isolates. (A) Correlations between antimicrobial MICs for 596 Pseudomonas aeruginosa clinical isolates. Spearman’s correlation matrix was plotted in R using the corrplot package. Positive correlations are displayed in blue and negative correlations are in red. The color intensity and size of the circle are proportional to the correlation coefficients. Correlation coefficients without statistical significance are not shown. (B) Venn diagram showing the overlap of antimicrobial nonsusceptibility among the clinical isolates. Numbers represent the number of isolates. PIP, piperacillin; TZP, piperacillin-tazobactam; CAZ, ceftazidime; CZA, ceftazidime-avibactam; CT, ceftolozane-tazobactam; FEP, cefepime; ATM, aztreonam; IPM, imipenem; IMR, imipenem-relebactam; MEM, meropenem; AMK, amikacin; CIP, ciprofloxacin; CL, colistin; NS, nonsusceptible.

There was a strong positive correlation between the MICs for CZA and ceftazidime (Spearman’s correlation coefficient rho = 0.82, *P < *0.001) but relatively weaker correlations between the CZA and imipenem MICs (Spearman’s rho = 0.53, *P < *0.001) among the 596 P. aeruginosa clinical isolates (Fig. 1A).

### CZA susceptibility among ceftazidime-nonsusceptible (CAZ-NS) and imipenem-nonsusceptible (IPM-NS) isolates.

Of the 596 P. aeruginosa isolates, there were 340 (57.1%) ceftazidime-susceptible (CAZ-S) and imipenem-susceptible (IPM-S) isolates, 98 (16.4%) CAZ-S but IPM-NS isolates, 36 (6.0%) IPM-S but CAZ-NS isolates, and 122 (20.5%) CAZ-NS and IPM-NS isolates (Fig. 1B; see Table S1 in the supplemental material).

In total, 26.5% (158/596) of P. aeruginosa isolates demonstrated nonsusceptibility to ceftazidime, 61.4% (97/158) of these were susceptible to CZA (Table 2 and Fig. 1B). Among the CAZ-NS subset, CZA produced similar susceptibility rates for IPM-NS isolates (61.5%, 75/122) and for IPM-S isolates (61.1%, 22/36), indicating that susceptibility to CZA was unrelated to the resistance profile of imipenem (Table 2). In addition, among the CAZ-NS subset, the median imipenem MIC was 16 mg/L for both 97 CZA-susceptible (CZA-S) isolates and 61 CZA-nonsusceptible (CZA-NS) isolates, suggesting low overlap between CZA resistance and imipenem resistance.

**TABLE 2:** Susceptibility rate of ceftazidime-avibactam against ceftazidime-nonsusceptible and imipenem-nonsusceptible P. aeruginosa isolates^*a*^

Phenotype	CAZ-S (%)	CAZ-NS (%)	Total (%)
IPM-S	100 (340/340)^*b*^	61.1 (22/36)	96.3 (362/376)
IPM-NS	100 (98/98)	**61.5 (75/122)**	78.6 (173/220)
Total	100 (438/438)	61.4 (97/158)	89.8 (535/596)

a The value in parenthesis is the ratio of ceftazidime-avibactam-susceptible isolates to the total number of corresponding phenotype isolates. The bold type aims to highlight CAZ-NS, IPM-NS but CZA-S isolates.

b CAZ, ceftazidime; IPM, imipenem; S, susceptible; NS, nonsusceptible.

While 36.9% (220/596) of P. aeruginosa isolates displayed nonsusceptibility to imipenem, 78.6% (173/220) of IPM-NS isolates were susceptible to CZA, including 98 (44.5%) CAZ-S isolates and 75 (34.1%) CAZ-NS isolates (Table 2).

For 122 CAZ-NS and IPM-NS isolates, susceptibility rates for CZA, CT, and IMR were 61.5% (75/122), 54.9% (67/122), and 51.6% (63/122), respectively.

### Mechanisms of resistance to ceftazidime among CAZ-NS, IPM-NS but CZA-S isolates.

**FIG 2:**
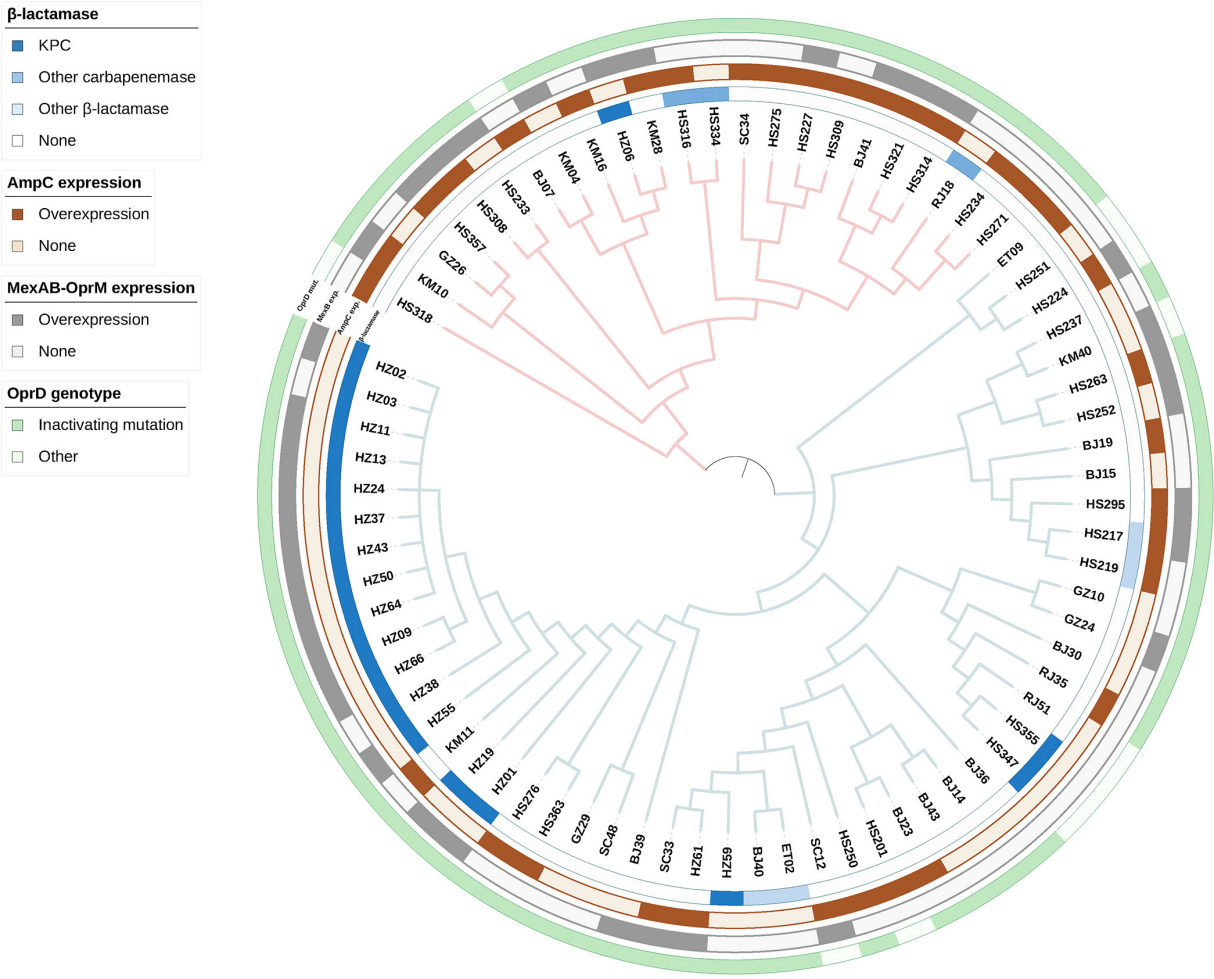
Resistance mechanisms of 75 ceftazidime-nonsusceptible, imipenem-nonsusceptible but ceftazidime-avibactam-susceptible Pseudomonas aeruginosa isolates. Phylogenetic analysis was constructed based on a core-genome alignment of 75 isolates from this study, with branch colors representing the clades. The outer rings are annotated with acquired β-lactamases, the expression level of chromosomal *ampC*, the expression level of chromosomal efflux pump *mexAB-oprM*, and the genotypic change of porin OprD.

To illuminate how avibactam overcomes ceftazidime resistance, mechanisms of ceftazidime nonsusceptibility were investigated using whole-genome sequencing (WGS) and quantitative real-time PCR (qRT-PCR) among 75 CAZ-NS, IPM-NS but CZA-S isolates (Fig. 2).

Among these 75 isolates, 26 (34.7%) carried horizontally acquired β-lactamases (Fig. 2) and manifested MICs for CZA ranging from 2 to 8 mg/L. Carbapenemases were present in 22 isolates: KPC-2 (*n* = 19), VIM-2 (*n* = 1), and GES-5 (*n* = 1), as well as coexistence of GES-5 and PER-1 (*n* = 1). The VIM-2-carrying isolate had a CZA MIC of 8 mg/L. Extended-spectrum β-lactamases (ESBL) were detected in four isolates (excluded the one coexisting with GES-5): PER-1 (*n* = 2) and OXA-101 (*n* = 1), as well as coexistence of PER-1 and OXA-101 (*n* = 1) (Table S2).

Furthermore, 34 isolates presented an *ampC* overexpression phenotype (Fig. 2). Changes in regulatory factors contributing to *ampC* overproduction (*ampR*, *ampD*, and *dacB*) were witnessed in 21 isolates, including a premature stop codon (*n* = 6) and amino acid substitution G154R (*n* = 3) in *ampR*, a frameshift mutation (*n* = 7) in *ampD*, and a premature stop codon (*n* = 1), frameshift mutation (*n* = 3), and amino acid substitution T428P (*n* = 1) in *dacB*. A total of 17 subtypes of PDC were present in the 75 isolates (Table S3), with PDC-8 (*n* = 18) being the most common, followed by PDC-5 (*n* = 16), PDC-1 (*n* = 8), and PDC-3 (*n* = 6). Additionally, overexpression of the efflux pump gene *mexAB*-*oprM* was detected in 35 isolates (Fig. 2).

Inactivating mutations in *oprD* associated with IPM resistance were observed in 65 (86.7%) isolates, including frameshift mutations (*n* = 53) and premature stop codons (*n* = 12) (Fig. 2).

### Mechanisms of ceftazidime nonsusceptibility among CAZ-NS, IPM-S, and CZA-S isolates.

Among the 22 CAZ-NS, IPM-S, and CZA-S strains, only one isolate carried an ESBL-encoding gene, *bla*_PER-1_. These isolates did not harbor carbapenemases, nor did they show inactivating mutations in *oprD*. In total, 59.1% (13/22) of isolates displayed *ampC* overexpression, and 50.0% (11/22) of isolates exhibited alteration of regulatory factors contributing to *ampC* overproduction, including a premature stop codon (*n* = 1), frameshift mutation (*n* = 1), and amino acid substitution D135N/Y (*n* = 4) in *ampR*, a premature stop codon (*n* = 4) and frameshift mutation (*n* = 1) in *ampD*, and a premature stop codon (*n* = 1) in *dacB*. A total of 10 subtypes of PDC were detected in these 22 isolates (Table S3), with PDC-3 (*n* = 10) being the most prevalent, followed by PDC-8 (*n* = 2), PDC-16 (*n* = 2), and PDC-63 (*n* = 2).

### Susceptibility to three β-lactam/β-lactamase inhibitor combinations for KPC-2-positive P. aeruginosa.

Among the 158 CAZ-NS isolates, 22 carried KPC-2 carbapenemase and did not carry metallo-β-lactamase. They were assigned to five STs, ST463 (*n* = 17), ST16 (*n* = 2), ST238 (*n* = 1), ST244 (*n* = 1), and an undefined ST (*n* = 1). Notably, their susceptibility rates to CZA, CT, and IMR were 86.4% (19/22), 0%, and 9.1% (2/22), respectively.

Furthermore, 95% (19/20) of IMR-nonsusceptible isolates had an inactivating mutation of the *oprD* gene; 17 of these isolates had premature stop codons in the amino acid at position 18, and two strains had mutations in loop 3, which is a passage channel within OprD for imipenem (13). The one remaining IMR-nonsusceptible isolate had an OprD sequence identical to that of P. aeruginosa type strain LESB58. In addition, one of the two IMR-susceptible isolates had an OprD sequence identical to that of LESB58, while the other IMR-susceptible isolate had a premature stop codon of OprD in the amino acid at position 327, located between loop 7 and loop 8, which are not involved in the passage of imipenem (13).

## DISCUSSION

This study showed that P. aeruginosa clinical isolates were highly susceptible to three β-lactam/β-lactamase inhibitor combinations, CZA, IMR, and CT. In total, 89.8% of P. aeruginosa clinical isolates from China were susceptible to CZA with MIC_50_/MIC_90_ of 2/16 mg/L, and avibactam restored the susceptibility to ceftazidime in 61.4% of CAZ-NS isolates. Similar results were found in a study in Spain, in which antimicrobial activity of CZA was demonstrated against 1,445 P. aeruginosa isolates, with a susceptibility rate of 94.2% and MIC_50_/MIC_90_ of 2/8 mg/L (14). The overall susceptibility of P. aeruginosa to IMR was 88.9%, and the addition of relebactam to imipenem conferred susceptibility to 70% of IPM-NS isolates in this study. Consistently, a high susceptibility rate to IMR (89.1%) was found for 2,623 P. aeruginosa isolates from Asia, and relebactam restored imipenem susceptibility from 0% to 64.6% for 805 IPM-NS isolates (9). Imipenem is a potent inducer of AmpC expression, and relebactam was shown to inhibit AmpC, which may account for synergy between imipenem and relebactam against P. aeruginosa (15, 16). High *in vitro* activity (88.9%) was also observed for CT in the 596 isolates, with an MIC_50_/MIC_90_ of 0.5/8 mg/L. Nevertheless, variation was noted in the susceptibility to CT in different regions, which ranged from 89.1% in Europe to 98.2% in North America (17). The high susceptibility to CT could be attributed to the enhanced affinity of ceftolozane to penicillin-binding proteins (PBPs) and its high stability for PDC enzymes in P. aeruginosa (15).

P. aeruginosa isolates showed low cross-resistance between ceftazidime and imipenem in this study, with 44.5% (98/220) of IPM-NS isolates being susceptible to ceftazidime. Susceptibility (32.5%, 389/1,198) to ceftazidime was also reported for carbapenem-resistant P. aeruginosa (CRPA), as evidenced by data from the Antimicrobial Testing Leadership and Surveillance (ATLAS) program in the Asia-Pacific region from 2015 to 2019 (18). Another American study found that 66.9% (101/151) of CRPA remained susceptible to ceftazidime (19).

In this study, CZA was superior to CT and IMR in combatting KPC-positive P. aeruginosa isolates (susceptibility rates, 86.4% versus 0% and 9.1%, respectively). A global study reported a susceptibility rate to CZA of 75.9% for 29 KPC-2-positive clinical P. aeruginosa isolates, while another group from China reported a CZA susceptibility rate of 49.7% for 151 KPC-2-positive clinical P. aeruginosa isolates (11, 20). Nonetheless, little is known about the activity of IMR against KPC-2-producing P. aeruginosa.

Among 22 isolates carrying KPC-2 carbapenemases alone, 95% (19/20) of IMR-nonsusceptible isolates had an inactivating mutation of the *oprD* gene. Porin OprD in the outer membrane of P. aeruginosa is the gate through which imipenem enters the periplasmic space. The inactivating mutation of the *oprD* gene makes this porin nonfunctional, which confers resistance to imipenem (21) and further IMR. However, OprD is not involved in the uptake of ceftazidime or avibactam (7). Additionally, avibactam is superior in inhibiting KPC-2 enzyme in comparison with relebactam (IC_50_ values, 10 nM versus 230 nM). Therefore, the combination of KPC-2 enzyme and nonfunctional porin OprD leads to IMR resistance in P. aeruginosa (especially in CRPA), and CZA is more active than IMR in fighting against KPC-2-producing P. aeruginosa.

In this study, CZA showed good activity against CAZ-NS and IPM-NS P. aeruginosa, supporting the clinical use of CZA in the treatment of infections caused by DTR-P. aeruginosa. We also found that 45.3% (34/75) of isolates demonstrated overexpression of AmpC in CAZ-NS and IPM-NS but CZA-S isolates, indicating that overexpressed AmpC could be inhibited by avibactam. Consistently, the addition of avibactam increased ceftazidime susceptibility for 46 AmpC-hyperproducing clinical isolates from 10.9% to 76.1% (7). Similar findings were reported for a collection of PAO1 isogenic mutants with multiple levels of AmpC hyperproduction due to regulatory factor mutations (*ampD*, *ampDh2*, *ampDh3*, and *dacB*) (7).

A total of 20 subtypes of PDC were detected in 97 CAZ-NS but CZA-S isolates in the present study, indicating that these PDC structures are unrelated to CZA resistance. PDC has high sequence polymorphism with different spectrums of hydrolytic activity and has been classified into over 513 variants to date (https://arpbigidisba.com), few of which could contribute to CZA resistance (22). Previous studies reported that avibactam reversed the ceftazidime resistance engendered by most PDC enzymes in P. aeruginosa, but limited information is available about specific PDC subtypes (23). Hence, our result is complementary to the understanding of specific PDC subtypes which may be associated with CZA susceptibility.

The limitation of this study is that there are isolates with unknown mechanisms conferring resistance to ceftazidime in CAZ-NS but CZA-S isolates, and thus it is difficult to speculate on the mechanism by which avibactam reversed resistance.

In conclusion, our results elucidated that three antipseudomonal β-lactam/lactamase combinations (CZA, CT, and IMR) were active against the majority of P. aeruginosa isolates. CZA remained active against CAZ-NS and IPM-NS P. aeruginosa isolates primarily by inhibiting KPC-2 enzyme and overproduced AmpC. CZA was more active than IMR in fighting against KPC-2-producing P. aeruginosa, because the combination of KPC-2 and nonfunctional porin OprD resulted in IMR resistance.

## MATERIALS AND METHODS

### Bacterial isolates.

A total of 596 nonduplicate clinical P. aeruginosa isolates were collected from 11 hospitals across China from July 2018 and February 2019 (Table S4) (24). The 596 P. aeruginosa isolates were predominantly from respiratory tract specimens (61.7%), followed by urinary tract (12.6%) and blood specimens (4.0%).

### Antimicrobial susceptibility testing.

Antimicrobial susceptibility was tested by the agar dilution method, except that antimicrobial susceptibility to colistin was tested by the broth microdilution method. Susceptibility to all antimicrobial agents was interpreted according to the Clinical and Laboratory Standards Institute (CLSI) breakpoints (25), except that colistin MICs were interpreted using EUCAST breakpoints for P. aeruginosa (susceptible, ≤4 mg/L; resistant, >4 mg/L) (www.eucast.org). P. aeruginosa ATCC 27853 served as a quality control strain.

### Whole-genome sequencing (WGS) and bioinformatic analysis.

The CAZ-NS isolates were sent for WGS performed with an Illumina NovaSeq system. The raw reads were assembled using SPAdes (https://github.com/ablab/spades) after trimming. The presence of resistance genes was explored using the Comprehensive Antibiotic Resistance Database (CARD) webtool (https://card.mcmaster.ca/home). Multilocus sequence typing (MLST) was ascertained according to the guideline on P. aeruginosa on the MLST website (https://pubmlst.org/organisms/pseudomonas-aeruginosa). A core genome phylogenetic tree was generated using kSNP and visualized using iTOL (26, 27). Sequence analysis of porin *oprD*, *ampC*, and its regulatory factors (*ampR*, *ampD*, and *dacB*) was performed after extraction from a fasta file.

### Quantitative real-time PCR (qRT-PCR).

The transcription levels of the gene *ampC* encoding P. aeruginosa AmpC and efflux pump-encoding genes (*mexB*, *mexC*, *mexE*, and *mexY*) were determined by qRT-PCR for clinical isolates as described previously (28). Briefly, the extraction of total RNA was performed with a TaKaRa miniBEST universal RNA extraction kit, and the synthesis of cDNA was accomplished with SMART MMLV reverse transcriptase. The transcription levels were measured with a PrimeScript reverse transcription-PCR kit as recommended by the manufacturers, with housekeeping gene *rpsL* as the internal reference. Isolates were considered to overexpress AmpC, MexAB-OprM, MexCD-OprJ, MexEF-OprN, and MexXY-OprM when the transcriptional levels of *ampC*, *mexB*, *mexC*, *mexE*, and *mexY* were at least 10-, 3-, 10-, 10-, and 10-fold higher, respectively, than those of the PAO1 strain (28).

### Statistical analysis.

Data were analyzed and visualized using R software v4.1.3. Spearman rank-order correlation was used to evaluate associations between antibiotic MICs.

### Data availability.

The genomic sequence data have been submitted to the NCBI GenBank database with BioProject number PRJNA891673.
