# Supplementary material for: Comparison of In Vitro Activity of Ceftazidime-Avibactam and Imipenem-Relebactam against Clinical Isolates of Pseudomonas aeruginosa
Source: Microbiol Spectr. 2023 May 18;11(3):e00932-23. doi: 10.1128/spectrum.00932-23 (PMC10269746; doi:10.1128/spectrum.00932-23)
Supplement: Supplemental file 1 — Tables S1-S4. Download spectrum.00932-23-s0001.docx, DOCX file, 0.02 MB [file spectrum.00932-23-s0001.docx]

**Table S1. Ceftazidime- and imipenem-susceptibility profiles among 596 *P. aeruginosa* isolates.**

| **Phenotype  n (%)** | **CAZ-S** | **CAZ-NS** | **Total** |
| --- | --- | --- | --- |
| **IPM-S** | 340 (57.1%) | 36 (6.0%) | 376 (63.1%) |
| **IPM-NS** | 98 (16.4%) | 122 (20.5%) | 220 (36.9%) |
| **Total** | 438 (73.5%) | 158 (26.5%) | 596 (100%) |

Abbreviation: CAZ, ceftazidime; IPM, imipenem; S, susceptible; NS, nonsusceptible.

**Table S2. Resistance mechanisms in 75 ceftazidime-nonsusceptible, imipenem-nonsusceptible but ceftazidime-avibactam-susceptible *P. aeruginosa* isolates.**

| **Resistance mechanisms ^a^** | **Number of isolates** |
| --- | --- |
| **Acquired β-lactamase (n = 26)** | |
| **Carbapenemase positive (n = 22)** | |
| KPC-2 | 19 |
| VIM-2 | 1 |
| GES-5 | 1 |
| GES-5, PER-1 | 1 |
| **ESBL positive, carbapenemase negative (n = 4)** | |
| PER-1 | 2 |
| OXA-101 | 1 |
| PER-1 and OXA-101 | 1 |
| **No acquired β-lactamases detected (n = 49)** | |
| AmpC overexpression | 13 |
| AmpC + MexAB overexpression | 11 |
| AmpC + MexAB + MexEF overexpression | 1 |
| AmpC + MexAB + MexXY overexpression | 2 |
| AmpC + MexEF overexpression | 2 |
| AmpC + MexXY overexpression | 2 |
| MexAB overexpression | 5 |
| MexAB + MexEF + MexXY overexpression | 1 |
| MexEF overexpression | 3 |
| Unknown | 9 |

Abbreviation: ESBL, extended-spectrum β-lactamase.

**Table S3. Subtypes of *Pseudomonas*-derived cephalosporinase AmpC (PDC) in 97 ceftazidime-nonsusceptible but ceftazidime-avibactam-susceptible *Pseudomonas aeruginosa* isolates.**

| Subtypes of PDC | CAZ-NS and IPM-NS  (n = 75) | CAZ-NS but IPM-S  (n = 22) | Total  (n = 97) |
| --- | --- | --- | --- |
| PDC-8 | 18 | 2 | 20 |
| PDC-5 | 16 | 1 | 17 |
| PDC-3 | 6 | 10 | 16 |
| PDC-1 | 8 | 0 | 8 |
| PDC-24 | 4 | 1 | 5 |
| PDC-39 | 4 | 1 | 5 |
| PDC-30 | 3 | 1 | 4 |
| PDC-16 | 1 | 2 | 3 |
| PDC-11 | 2 | 0 | 2 |
| PDC-127 | 2 | 0 | 2 |
| PDC-22 | 2 | 0 | 2 |
| PDC-35 | 2 | 0 | 2 |
| PDC-36 | 2 | 0 | 2 |
| PDC-38 | 2 | 0 | 2 |
| PDC-63 | 0 | 2 | 2 |
| PDC-14 | 1 | 0 | 1 |
| PDC-31 | 1 | 0 | 1 |
| PDC-34 | 0 | 1 | 1 |
| PDC-37 | 1 | 0 | 1 |
| PDC-97 | 0 | 1 | 1 |

Abbreviation: CAZ, ceftazidime; IPM, imipenem; S, susceptible; NS, nonsusceptible.

**Table S4.** **Clinical *P. aeruginosa* isolates collected from multiple medical centers in China (n = 596).**

| Hospital | Province or municipality | Region | Isolation date  (mm/yyyy) | Number of isolates |
| --- | --- | --- | --- | --- |
| BD | Beijing | North China | 07/2018–11/2018 | 50 |
| BJ | Beijing | North China | 08/2018–01/2019 | 47 |
| ET | Shanghai | East China | 07/2018–09/2018 | 18 |
| HS | Shanghai | East China | 07/2018–09/2018 | 163 |
| RJ | Shanghai | East China | 12/2018–02/2019 | 47 |
| GZ | Guangdong | South Central China | 10/2018–02/2019 | 50 |
| HZ | Zhejiang | East China | 09/2018 | 60 |
| JL | Jilin | Northeast China | 08/2018–11/2018 | 49 |
| GS | Gansu | Northwest China | 11/2018 | 14 |
| KM | Yunnan | Southwest China | 08/2018–10/2018 | 49 |
| SC | Sichuan | Southwest China | 11/2018–02/2019 | 49 |
